# *SCN5A* mutation G615E results in Na_V_1.5 voltage-gated sodium channels with normal voltage-dependent function yet loss of mechanosensitivity

**DOI:** 10.1080/19336950.2019.1632670

**Published:** 2019-07-02

**Authors:** Peter R. Strege, Arnaldo Mercado-Perez, Amelia Mazzone, Yuri A. Saito, Cheryl E. Bernard, Gianrico Farrugia, Arthur Beyder

**Affiliations:** a Enteric NeuroScience Program, Division of Gastroenterology and Hepatology, Mayo Clinic, Rochester, MN, USA; b Medical Scientist Training Program (MSTP), Mayo Clinic, Rochester, MN, USA; c Department of Physiology and Biomedical Engineering, Mayo Clinic, Rochester, MN, USA

**Keywords:** Electrophysiology, mechanotransduction, ion channel, voltage-gated sodium channel type 5, functional gastrointestinal disorder, irritable bowel syndrome

## Abstract

*SCN5A* is expressed in cardiomyocytes and gastrointestinal (GI) smooth muscle cells (SMCs) as the voltage-gated mechanosensitive sodium channel Na_V_1.5. The influx of Na^+^ through Na_V_1.5 produces a fast depolarization in membrane potential, indispensable for electrical excitability in cardiomyocytes and important for electrical slow waves in GI smooth muscle. As such, abnormal Na_V_1.5 voltage gating or mechanosensitivity may result in channelopathies. *SCN5A* mutation G615E – found separately in cases of acquired long-QT syndrome, sudden cardiac death, and irritable bowel syndrome – has a relatively minor effect on Na_V_1.5 voltage gating. The aim of this study was to test whether G615E impacts mechanosensitivity. Mechanosensitivity of wild-type (WT) or G615E-Na_V_1.5 in HEK-293 cells was examined by shear stress on voltage- or current-clamped whole cells or pressure on macroscopic patches. Unlike WT, voltage-clamped G615E-Na_V_1.5 showed a loss in shear- and pressure-sensitivity of peak current yet a normal leftward shift in the voltage-dependence of activation. In current-clamp, shear stress led to a significant increase in firing spike frequency with a decrease in firing threshold for WT but not G615E-Na_V_1.5. Our results show that the G615E mutation leads to functionally abnormal Na_V_1.5 channels, which cause disruptions in mechanosensitivity and mechano-electrical feedback and suggest a potential contribution to smooth muscle pathophysiology.

## Introduction

The voltage-gated mechanosensitive Na^+^ channel Na_V_1.5 is expressed by *SCN5A* in human cardiac myocytes and gastrointestinal (GI) smooth muscle cells (SMCs) [1,2]. *SCN5A* mutations are well established to cause cardiac conduction disorders, called channelopathies. Interestingly, patients with *SCN5A* cardiac channelopathies have an increased prevalence of irritable bowel syndrome (IBS) [3], and conversely, functionally abnormal *SCN5A* mutations are present in 2–3% of IBS patients [4–6].

Mechanosensitivity is important for the function of all cells [7], but it plays an especially important role in organ systems whose primary function is mechanical, such as cardiovascular, gastrointestinal, and urinary. In electrically excitable systems, mechanical forces regulate function by mechano-electrical feedback [8]. For example, mechanical stretch of neurons reversibly depolarizes the resting membrane potential and increases the frequency of action potentials [9], which is balanced by mechanosensitive voltage-gated potassium channels that provide a “mechanical brake” to their excitability [10]. Na_V_1.5 channels are gated by voltage, but they are also mechanosensitive [11,12], and mechanosensitivity of these channels is particularly relevant because they are expressed in heart and gut, which are mechanically active organs. Indeed, disruptions in Na_V_1.5 mechanosensitivity may contribute to cardiac conduction disorders [13]. In cardiomyocytes (7), GI SMCs [14,15], and heterologous expression systems [11,12,16], mechanical stimuli alter Na_V_1.5 function by increasing peak Na^+^ current (I_PEAK_), hyperpolarizing the voltage dependence of activation (V_1/2A_) and availability (inactivation, V_1/2I_), and accelerating channel kinetics. However, the mechanism of Na_V_1.5 mechanosensitivity remains unclear, which limits our ability to understand the contributions of Na_V_1.5 mutations to pathophysiology.

Previous work showed that disease-associated Na_V_1.5 mutations can disrupt voltage-gating, and a portion also disrupts mechanosensitivity [5,6]. Testing whether any Na_V_1.5 mutation could affect these two functions separately may illuminate the relationship between these functions. The mutation G615E Na_V_1.5 was found in several studies to associate with cardiac conduction disorders [17–20] and irritable bowel syndrome [4] but does not appear to lead to significant disruptions in Na_V_1.5 voltage-dependent function. In this study, we compare the mechanosensitivities of wild-type (WT) and G615E Na_V_1.5, a missense mutation in the DI-DII linker with normal current density [4] but a potentially disrupted mechanosensitivity [21].

## Methods

### Molecular biology

#### Plasmids

A single nucleotide change (c.1844 G→A) was engineered by site-directed mutagenesis in a construct containing the most common splice variant of *SCN5A* (hH1c1, H558/Q1077del) to substitute G615E in the Na^+^ channel α-subunit (p.G615E-Na_V_1.5) using the QuikChange II XL Site-Directed Mutagenesis Kit. The integrity of the construct and the presence of the desired mutation were verified by DNA sequencing.

#### Heterologous expression and cell culture

Wild-type (WT) Na_V_1.5 or p.G615E-Na_V_1.5 (G615E Na_V_1.5) were co-transfected with pEGFP-C1 into HEK-293 cells using Lipofectamine 2000 (Thermo Fisher Scientific, Massachusetts, USA).

### Electrophysiology

#### Pipette fabrication and data acquisition

Electrodes were pulled to a resistance of 2–5 MΩ from KG12 (Kimble glass, Fisher Scientific, Massachusetts, USA) for whole-cell voltage- or current-clamp or to a resistance of 1–2 MΩ from Garner 8250 glass for cell-attached pressure-clamp on a P97 puller (Sutter Instruments, California, USA) and coated with heat-cured R6101 polymer (Dow Corning, MI). Whole-cell and cell-attached patch data from HEK-293 cells were recorded at 20 kHz with an Axopatch 200B patch clamp, CyberAmp320, Digidata 1550, and pClamp 10.5 software (Molecular Devices, California, USA).

#### Whole-cell voltage clamp

The intracellular solution contained (in mM): 135 K^+^, 130 CH_3_SO_3_^−^, 20 Cl^−^, 5 Na^+^, 5 Mg^2+^, 5 HEPES, 2 EGTA; pH 7.0, 290 mmol/kg. The extracellular solution contained (in mM): 15 Na^+^, 140 Cs^+^, 160 Cl^−^, 2.5 Ca^2+^, 5 K^+^, 10 HEPES, 5.5 glucose; pH 7.35, 305 mmol/kg. *Episodic protocol*. To measure peak Na^+^ current density, cells transfected with WT- or G615E-Na_V_1.5 were held at −120 mV before pulsed through a 2-stage, 24-step voltage ladder (1) from −80 to +35 mV in 5 mV intervals for 50 ms each and (2) to −30 mV for 50 ms. The times between sweeps and each of 10 runs were 250 ms and 6 s, respectively. Peak currents at each voltage step were normalized to the cell capacitance (pF) dialed in during recording or to the maximum peak inward current without shear. *Mechanical stimulation by shear stress*. Flow of extracellular (bath) solution was applied by gravity drip, calibrated to a rate of 10 mL/min with intravenous tubing.

#### Whole-cell current clamp

The intracellular solution contained (in mM): 135 K^+^, 130 CH_3_SO_3_^−^, 20 Cl^−^, 5 Na^+^, 5 Mg^2+^, 5 HEPES, 2 EGTA; pH 7.0, 290 mmol/kg. The extracellular solution contained (in mM): 150 Na^+^, 160 Cl^−^, 5 K^+^, 2.5 Ca^2+^, 10 HEPES, 5.5 glucose; pH 7.35, 305 mmol/kg. *Gap-free protocol*. To measure the change in frequency of spontaneous events, cells transfected with WT- or G615E-Na_V_1.5 were recorded continuously below the predicted threshold. Briefly, window currents were plotted automatically from whole-cell Na^+^ currents recorded in voltage-clamp mode. With the range of the window current calculated to determine the threshold to elicit membrane potential spikes, the amplifier was switched to I-clamp mode, and current was continuously injected to hyperpolarize the membrane potential approximately 10–20 mV negative from the window current. Spontaneous activity was recorded with a gap-free protocol. With current injected to keep the resting potential hyperpolarized relative to the half-point of the voltage-dependence of inactivation in order to ensure full availability (WT, −89.9 ± 3.8 mV vs. G615E, −91.4 ± 3.5 mV; n = 8, *P* = 0.49 by a two-tailed unpaired t-test), spontaneous spike frequencies without shear ranged from 0.1 to 1.0 Hz for 80–90% of all experiments (8 of 9 WT and 18 of 22 G615E); experiments with baseline frequencies outside this range were excluded from the analysis. *Episodic protocols*. To establish a prediction for the threshold of elicited activity, cells transfected with WT- or G615E-Na_V_1.5 were held at −15 pA before pulsed through a 9-step current ladder from −15 pA to +25 pA in 5 pA intervals for 50 ms each. The time between sweeps was 1 s. To measure the probability of firing potentials, cells were held at resting current (e.g., −15 pA) and pulsed through 20 repetitions of a five-stage protocol with 5.45 s between sweeps: (1) to above the predicted threshold (e.g., +0 pA) for 50 ms and back to rest for 500 ms, then (2–5) to below the predicted threshold (e.g., −5 pA) for 50 ms and back to rest for 500 ms. *Mechanical stimulation*. Shear stress was applied by flow of extracellular solution at 10 mL/min, as described above in voltage-clamp mode.

#### Cell-attached patch pressure clamp

The pipette solution contained (in mM): 150 Na^+^, 160 Cl^−^, 5 K^+^, 2.5 Ca^2+^, 10 HEPES, 5.5 glucose; pH 7.35, 305 mmol/kg. The bath solution contained (in mM): 15 Na^+^, 140 Cs^+^, 160 Cl^−^, 2.5 Ca^2+^, 5 K^+^, 10 HEPES, 5.5 glucose; pH 7.35, 305 mmol/kg. *Episodic protocol and mechanical stimulation by pressure*. Na^+^ currents in macroscopic patches were elicited by a sequence of paired voltage ladders (Figure 3(a)) with pressures up to −60 mmHg applied during each step of the second voltage ladder (Figure 3(b)).

### Data analysis

**Figure 1.**
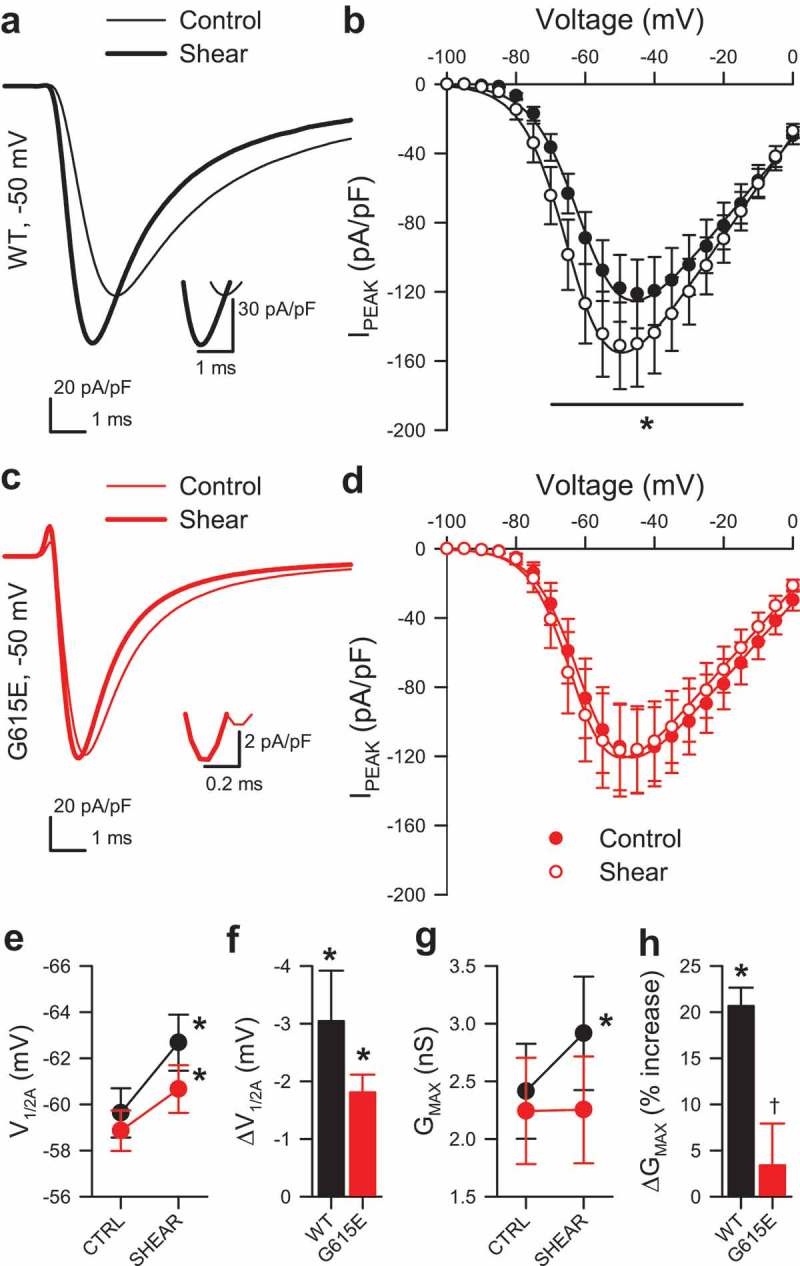
Shear sensitivity of voltage-gating of G615E Na_V_1.5 compared to WT Na_V_1.5. (a, c), Na^+^ current traces from wild-type (WT, *a*) or G615E (*c*) Na_V_1.5 channels elicited by a voltage step from −120 to −50 mV, before (–) or during shear stress (▬) by flow of extracellular solution at 10 mL/min. *Insets* show the current traces at peak. (b,d), Normalized peak current densities (I_PEAK_) of WT (b) or G615E (d) Na^+^ currents before (●) or during (○) shear stress. (e-f), Voltage of half-activation (V_1/2A_) during control or shear stress (e) and the average shift in V_1/2A_ with shear (f, ΔV_1/2A_) for WT (black) and G615E (red). (g-h), Peak conductance (G_MAX_) during control or shear stress (*g*) and the average change in G_MAX_ with shear (*h*, ΔG_MAX_) for WT (black) or G615E (red). (b,d,e,g), n = 12 cells each, *P < 0.05 shear *vs*. control and *P* < 0.05 interaction between genotype and shear by a three-way ANOVA with Tukey post-test. (f,h), n = 12 cells each, *P < 0.05% to 0% by a two-tailed one-sample t-test; †*P*< 0.05 to WT by a two-tailed unpaired t-test.

**Figure 2.**
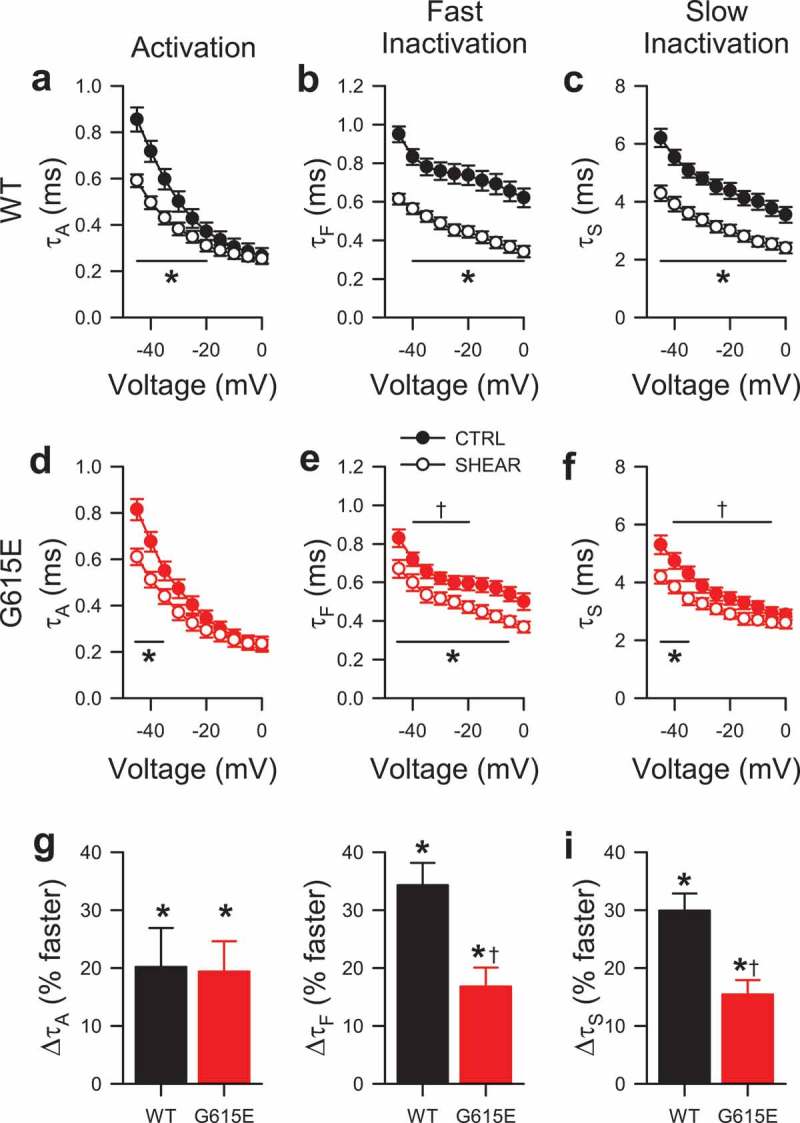
Shear sensitivity of voltage-dependent gating kinetics of G615E Na_V_1.5 compared to WT Na_V_1.5. (a-f), Time constants of activation (a, d; τ_A_) and two inactivation components (b-c, e-f) from wild-type (a-c, WT) or G615E (d-f) Na_V_1.5 currents, before (●) or during shear stress (○) by flow of extracellular solution at 10 mL/min [n = 12 cells each; *P < 0.05 shear stress *vs*. baseline controls and *P* < 0.05 interaction between voltage and shear (τ_A_) or between genotype and shear (τ_F_, τ_S_) by a three-way ANOVA with Tukey post-test; †*P*< 0.05 to WT by a two-tailed unpaired t-test]. (g-i), Average change in time constants of activation (g) and two inactivation components (h-i) of WT (black) or G615E (red) Na_V_1.5 currents at −30 mV (n = 12 cells each; *P < 0.05% to 0% by a two-tailed one-sample t-test; †*P* < 0.05 to WT by a two-tailed unpaired t-test).

**Figure 3.**
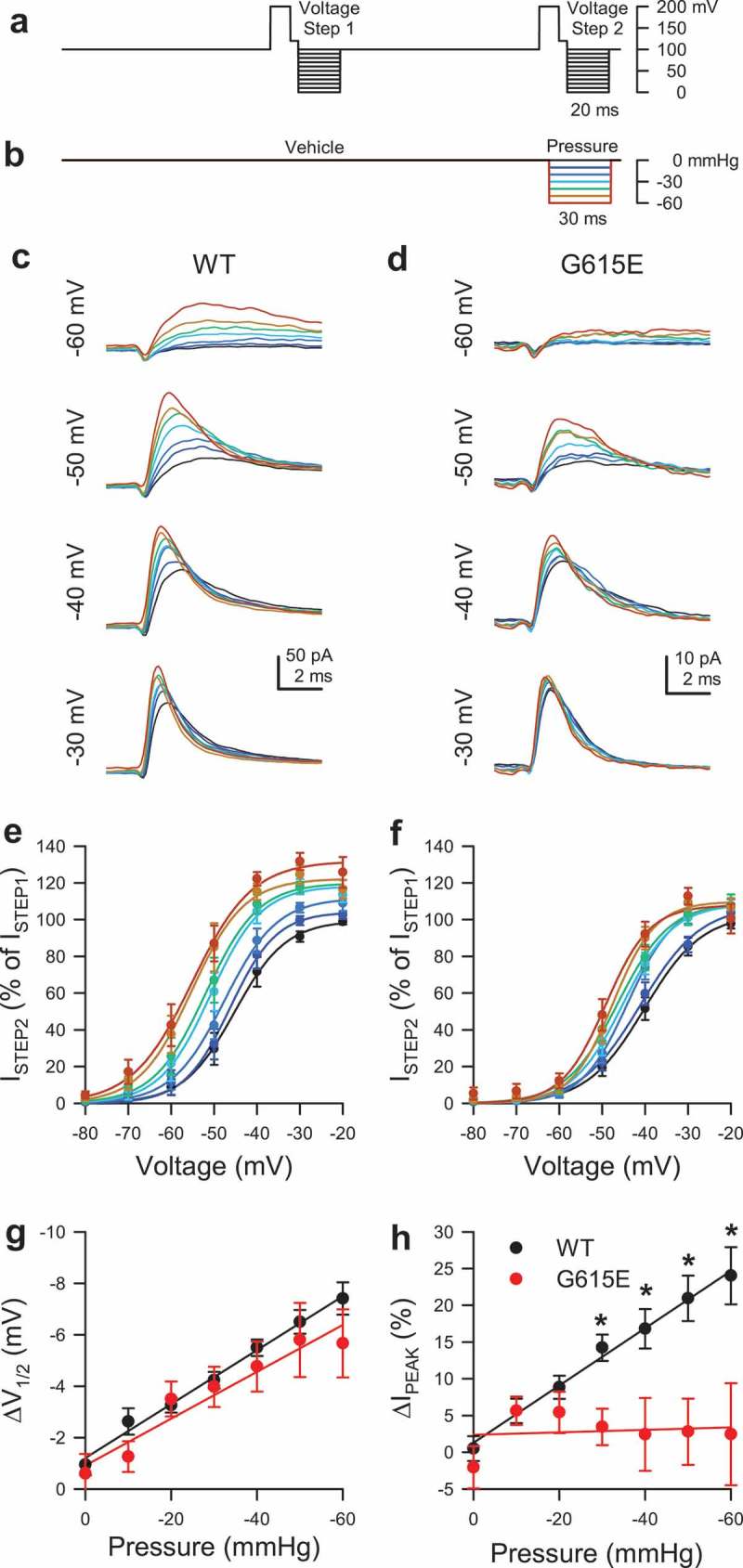
Pressure sensitivity of WT Na_V_1.5 compared to G615E Na_V_1.5. (a-b), Voltage-clamp (a) and pressure-clamp (b) protocols were used to elicit macroscopic Na^+^ currents. Pressure was off during voltage step 1 (vehicle) and on during step 2 (pressure). (c-d), Representative macroscopic patch currents in HEK293 cells transfected with WT- (c) or G615E- Na_V_1.5 (d), elicited by depolarizations to −60, −50, −40, or −30 mV during voltage step 2 with the pressure indicated by the color gradient defined in (b). (e-f), Steady-state activation curves of macroscopic patch currents for WT (e) or G615E-Na_V_1.5 (f) during voltage step 2 with the pressure indicated by the color gradient. *P* < 0.05 effect of pressure or voltage for WT- (d) and G615E-Na_V_1.5 (e) by a two-way ANOVA with Dunnett post-test. (g), Change in voltage of half-activation (ΔV_1/2A_) from WT (black) or G615E- Na_V_1.5 (red), the difference between V_1/2A_ during voltage step 2 and V_1/2A_ during step 1, plotted as a function of the pressure during step 2. *P* < 0.05 effect of pressure and *P* > 0.05 effect of genotype by a three-way ANOVA with Tukey post-test. (h), Change in peak current (ΔI_PEAK_) from WT (black) or G615E- Na_V_1.5 (red), the % increase in peak currents during voltage step 2 (I_STEP2_) normalized to same-sweep peak currents during step 1 (I_STEP1_), plotted as a function of the pressure during step 2. *P < 0.05 effect of pressure, genotype by a three-way ANOVA with Tukey post-test.

**Figure 4.**
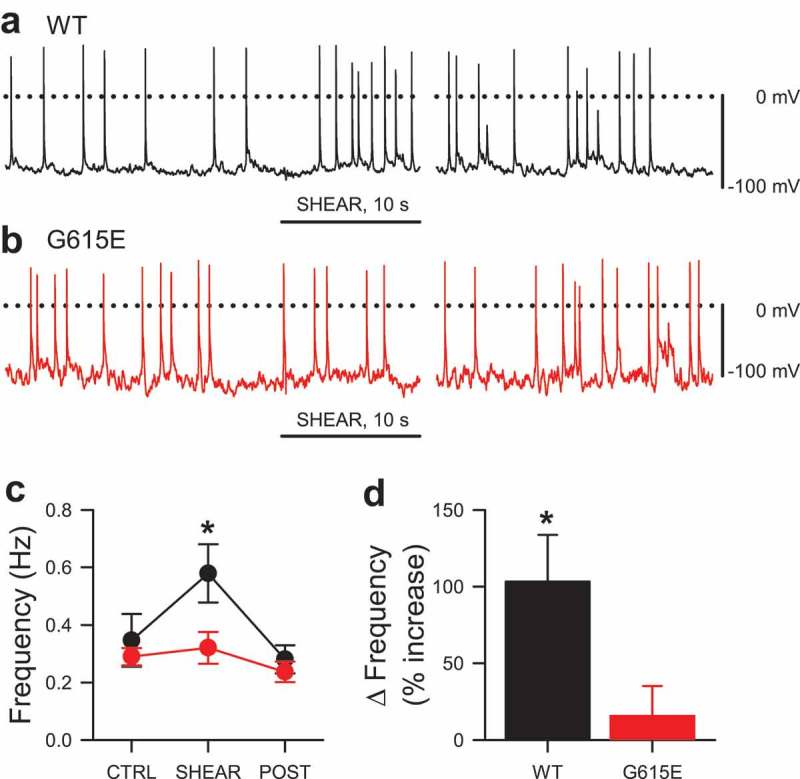
Mechanosensitivity of WT and G615E Na_V_1.5-induced spontaneous electrical excitability. (a-b), Gap-free current clamp recording of spontaneous potentials from an HEK293 cell expressing WT Na_V_1.5 (a) or G615E (b), before, during (underline), or 35 s after shear stress. (c), Average frequencies of spontaneous potentials from HEK293 cells expressing WT (black) or G615E (red) Na_V_1.5 channels, before (CTRL), during (SHEAR), or after (POST) shear stress (n = 6 cells each, *P < 0.05 *vs*. CTRL and *P* < 0.05 effect of genotype or shear by a two-way ANOVA with Dunnett post-test). (d), Average change in frequency of spontaneous potentials in HEK293 cells expressing WT (black) or G615E (red) Na_V_1.5 channels (n = 6 cells each, *P < 0.05 WT to 0% by a two-tailed one-sample t-test, NS for G615E).

**Figure 5.**
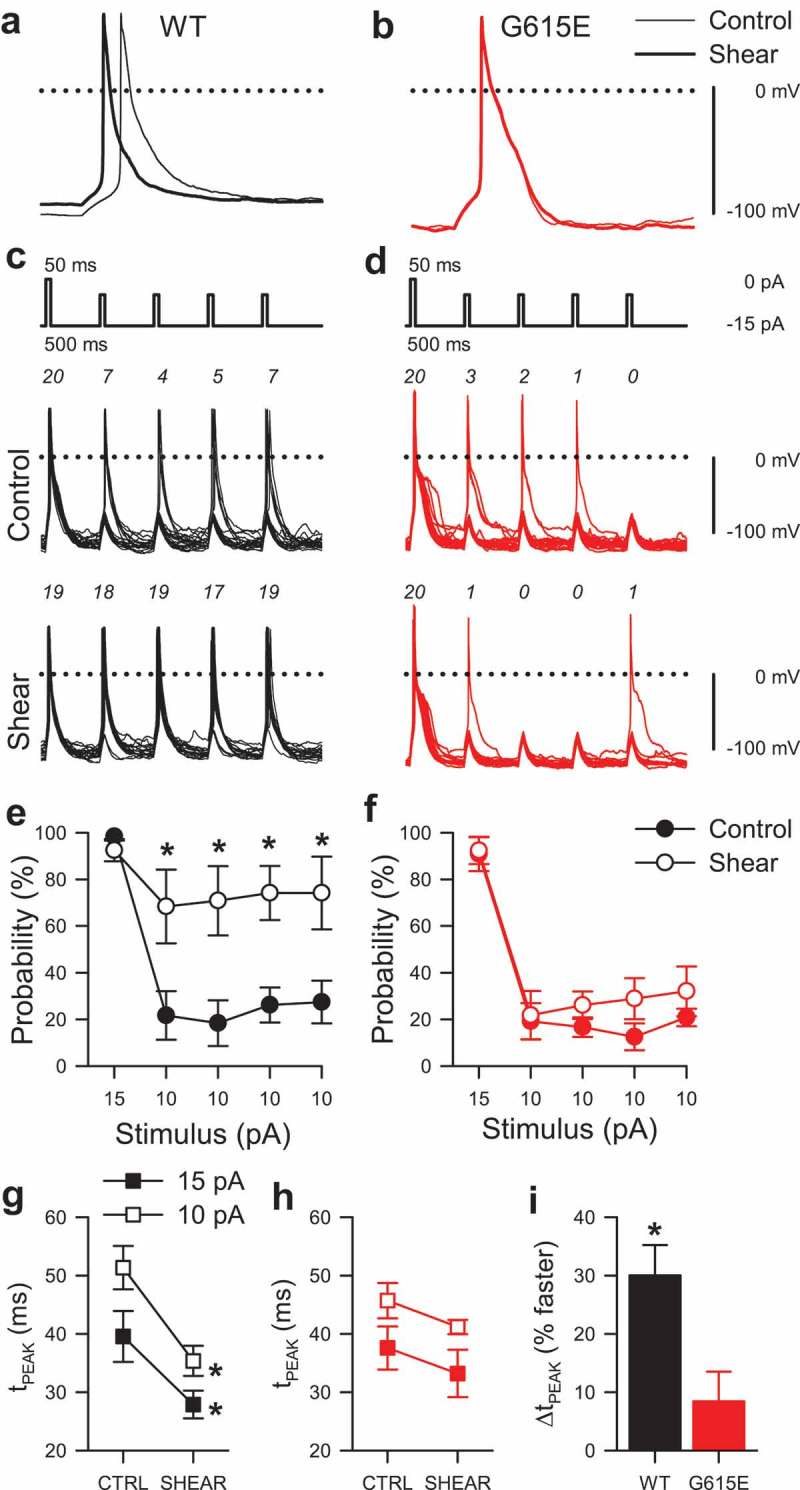
Mechanosensitivity of WT and G615E Na_V_1.5-induced elicited electrical excitability. (a-b), Potentials evoked by a 50-ms current stimulus before (–) or during (▬) shear stress from HEK293 cells expressing either WT (a) or G615E (b) Na_V_1.5 channels. (c-d), 20 overlapping sweeps of potentials from WT (c) or G615E-Na_V_1.5 (d) transfected HEK cells, induced by a current stimulus protocol (*inset*) with one super-threshold (Δ15 pA) 50-ms stimulus followed by four sub-threshold 50-ms stimuli (Δ10 pA), before (control) or during (shear) mechanical stimulation. (e-f), Fraction of potentials from WT (e) or G615E-Na_V_1.5 (f) transfected HEK cells evoked at the super-threshold 15-pA stimulus or four sub-threshold 10-pA stimuli before (●) or during (○) application of shear stress (n = 6 cells each; *P < 0.05 WT control *vs*. WT shear and *P* < 0.05 interaction between stimulus, genotype, and shear by a three-way ANOVA with Tukey post-test). (g-h), Time to peak potential (t_PEAK_) from cells expressing WT (g) or G615E-Na_V_1.5 (h) channels evoked at the 15-pA (■) or 10-pA (□) stimulus before (CTRL) or during shear stress (SHEAR) (n = 6 cells each; *P < 0.05, WT control *vs*. WT shear; *P* < 0.05, effect of stimulus or effect of shear; and *P* < 0.05, interaction between genotype and shear by a three-way ANOVA with Tukey post-test). (i), Average change in time to peak potential (Δt_PEAK_) induced by shear for WT (black) or G615E-Na_V_1.5 (red) (n = 6 cells each; *P < 0.05% to 0% by a two-tailed one-sample t-test).

To calculate whole-cell conductance and voltage dependence of activation, Na_V_1.5 current-voltage (I-V) plots were fit with the equation: I_V_ = G_MAX_*(V-E_REV_)/(1 + e^(V-V1/2A)/slope^), in which G_MAX_ is the maximum conductance of peak Na^+^ current, and V_1/2A_ is the voltage of half-activation. To calculate frequency, the number of spontaneous potentials firing past 0 mV in gap-free mode were expressed as a fraction of the 60- to 120-s acquisition time (Hz). To calculate the probability of firing, the number of evoked potentials firing past 0 mV were expressed as a fraction of the number of current stimuli. Response to pressure was measured by the change in peak Na^+^ current, I_STEP2_ – I_STEP1_ = ΔI_PEAK_. I–V curves were examined for any shifts in the V_1/2_ of activation (ΔV_1/2A_) versus paired controls without applied pressure. Data are expressed as the mean ± standard error of the mean (SEM). Change as a result of shear stress or pressure was assigned when *P* < 0.05 for mechano-stimulus to control by a one-sample t-test (Figure 1(f,h); Figure 2(g-i); Figure 4(d); Figure 5(i)), *P* < 0.05 for G615E to WT by a two-way ANOVA with Dunnett post-test (Figure 3(e-f), Figure 4(c)), or *P* < 0.05 for G615E to WT by a three-way ANOVA with Tukey post-test (Figure 1(b,d); Figure 2(a-f); Figure 3(g-h); Figure 5(e-h)).

## Results

### Effect of mechanical stimulation by shear stress on whole-cell WT and G615E Na_V_1.5 currents

In the absence of shear stress, we found that voltage-dependent peak conductance of G615E Na_V_1.5 was not different than WT Na_V_1.5 (G_MAX_ 2.41 ± 0.41 nS, WT *vs*. 2.24 ± 0.46 nS, G615E; n = 12, *P* > 0.05) (Figure 1(a,c,g)). As previously described [6,16], whole-cell Na_V_1.5 conductance increased in response to shear stress (G_MAX_ of WT Na_V_1.5: 2.41 ± 0.41 nS control to 2.91 ± 0.49 nS shear, 20.7 ± 2.0% increase, n = 12, *P < 0.001 to control) (Figure 1(a-b, g-h)). In contrast, G615E Na_V_1.5 peak current was unchanged by shear stress (G_MAX_: 2.24 ± 0.46 nS control to 2.25 ± 0.46 nS shear, 3.4 ± 4.5% change, n = 12, *P* > 0.05 control vs. shear) (Figure 1(c-d), (g-h)). Both WT and G615E Na_V_1.5 showed similar small but significant left-shifts of the half-points of voltage-dependence of activation (V_1/2A_) with shear stress (WT: −59.6 ± 1.1 mV control, −62.7 ± 1.2 mV shear; −3.0 ± 0.9 mV change in V_1/2A_; n = 12, *P* < 0.01; G615E: −58.9 ± 0.9 mV control, −60.7 ± 1.0 mV shear, −1.8 ± 0.3 mV change in V_1/2A_; n = 12, *P* < 0.01) (Figure 1(b, d-f)).

Having observed an acceleration in the time of peak current (insets, Figure 1(a,c)), we next examined WT and G615E Na^+^ currents for shear-induced changes to kinetics. Difference currents were constructed by subtracting composites of 12 families of whole-cell Na^+^ current during shear from the composites of their respective controls (Supplementary Figure 1). The negative deflection in the difference currents from WT was roughly twice the size than that from G615E, while the positive deflections were similar. Examining changes to parameters of Na^+^ current kinetics in greater detail, we found that the time constants of activation (τ_A_) were similarly faster for WT (+20.2 ± 6.7%, Figure 2(a)) and G615E (+19.4 ± 5.2%, Figure 2(d); n = 12, *P* < 0.05 by a one-sample t-test, *P* > 0.05 WT vs. G615E by a two-tailed unpaired t-test, Figure 2(g)); additionally, the time constants of fast (τ_F_) and slow inactivation (τ_S_) each were faster for both WT (τ_F_, +34.3 ± 3.8%; τ_S_, +29.9 ± 3.0%) and G615E channels (τ_F_, +16.8 ± 3.3%; τ_S_, +15.4 ± 2.5%; n = 12, *P* < 0.05 by one-sample t-tests, Figure 2(h-i)), as shown previously for WT [6,16]. However, shear-induced acceleration of inactivation in G615E was relatively less than in WT (Figure 2(h-i)), which may be because G615E control currents already inactivated faster than WT control currents (τ_F_ at −30 mV: WT, 0.76 ± 0.05 vs. G615E, 0.62 ± 0.03 ms; τ_S_ at −30 mV: WT, 4.81 ± 0.21 vs. G615E, 3.87 ± 0.23 ms; n = 12, *P* < 0.05 WT vs. G615E by two-tailed unpaired t-tests) (Figure 2(b-c, e-f, g-h)).

### Effect of pressure on WT and G615E Na_V_1.5 current within a patch

Next, we used another technique to confirm the loss of G615E Na_V_1.5 mechanosensitivity we found with shear stress. We examined WT and G615E Na_V_1.5 by simultaneous voltage- and pressure-clamp with on-cell patches [16,22]. With a two-step protocol to determine Na_V_1.5 pressure dependence [22] (Figure 3(a-b)), the currents elicited by “Voltage Step 1” test the voltage-dependence of Na_V_1.5, while currents from “Voltage Step 2” test the effect of pressure concurrently with voltage. We found that during Voltage Step 1 (0 mmHg), peak Na^+^ currents and voltage-dependence of activation were not statistically different between constructs (I_MAX_: WT, 71.6 ± 15.0 pA *vs*. G615E, 39.0 ± 12.8 pA, n = 9, *P* = 0.12; V_1/2A_: WT, 41.2 ± 4.5 mV *vs*. G615E, 39.4 ± 1.7 mV, n = 9, *P* = 0.71 WT to G615E by two-tailed unpaired t-tests). However, as with shear stress, there were significant differences in the responses of WT and G615E Na_V_1.5 to pressure (Table 1, Figure 3(c-f)). WT Na_V_1.5 V_1/2A_ was hyperpolarized proportionately with pressure, for a slope of −1.0 ± 0.1 mV per −10 mmHg (*P* < 0.05 effect of pressure by a three-way ANOVA with Tukey post-test) (Figure 3(e,g)), and WT Na_V_1.5 macroscopic peak currents increased proportionately with negative patch pressure, for a rate of 3.9 ± 0.7% per −10 mmHg (n = 10 cells, *P* < 0.05 effect of pressure, voltage, and interaction by a two-way ANOVA with Dunnett post-test) (Figure 3(e,h)). G615E Na_V_1.5 V_1/2A_ left-shifted with pressure (*P* < 0.05 effect of pressure and *P* > 0.05 effect of genotype) (Figure 3(f-g)), and the slope of ΔV_1/2A_ for G615E Na_V_1.5 did not differ from WT Na_V_1.5, −0.9 ± 0.3 mV per −10 mmHg (Figure 3(g), Table 1). In contrast to WT Na_V_1.5, peak Na^+^ currents of G615E did not respond to pressure, for a rate of only 0.2 ± 1.0% per −10 mmHg (n = 10 cells, *P* < 0.05 effect of genotype, *P* < 0.05 effect of interaction between genotype and pressure by a three-way ANOVA with Tukey post-test) (Figure 3(h), Table 1). In all, our data in both whole cell with shear stress and patch with pressure show that G615E Na_V_1.5, unlike WT Na_V_1.5, lacks substantial force-dependent changes in mechanically induced peak currents and kinetics, whilst retaining the responses in voltage-dependence.

**Table 1.** Effect of negative patch pressure on the change in peak Na^+^ current (ΔI_PEAK_) or the change in voltage of half-activation (ΔV_1/2A_) of WT or G615E Na_V_1.5.

Pressure (mmHg)	ΔI_PEAK_ (%)		ΔV_1/2A_ (mV)	
	WT	G615E	WT	G615E
−00	0.5 ± 0.2	−2.1 ± 2.9	−0.9 ± 0.4	−0.6 ± 0.8
−10	5.6 ± 1.7	5.6 ± 1.9	−2.6 ± 0.5	−1.3 ± 0.6
−20	8.8 ± 1.6	5.4 ± 2.8	−3.3 ± 0.3	−3.5 ± 0.7
−30	14.2 ± 0.2	3.5 ± 2.5*	−4.2 ± 0.3	−4.0 ± 0.8
−40	16.8 ±2.7	2.4 ± 5.0*	−5.5 ± 0.3	−4.8 ± 1.0
−50	20.9 ± 3.1	2.8 ± 4.5*	−6.5 ± 0.5	−5.8 ± 1.4
−60	24.0 ± 0.4	2.4 ± 6.9*	−7.4 ± 0.6	−5.7 ± 1.3

### Effect of shear stress on cell electrical excitability

Since Na_V_1.5 is involved in electrical excitability in the heart and gut, we pursued the effects of mechanical stimulation on Na_V_1.5 function in current clamp. Recent studies show that electrical excitability can be re-created in mammalian cell lines commonly used for heterologous expression, such as CHO and HEK-293 cells (14, 15). Therefore, we examined the spontaneous spiking of WT or G615E-transfected Na_V_1.5 HEK-293 cells for changes during shear stress in current-clamp mode (Figure 4(a-b)). For WT Na_V_1.5, we saw that shear stress reversibly increased the probability of spontaneous spiking events (Figure 4(a-b)); however, for G615E Na_V_1.5, shear stress failed to produce an increase in spiking frequency (WT Na_V_1.5: 0.35 ± 0.09 Hz control, to 0.58 ± 0.10 Hz shear, to 0.28 ± 0.05 Hz recovery, n = 8, *P* < 0.05 control vs. shear; G615E Na_V_1.5: 0.29 ± 0.03 Hz control, to 0.32 ± 0.06 Hz shear, to 0.24 ± 0.04 Hz recovery, n = 18; *P* < 0.05 effect of genotype and shear; *P* < 0.05 interaction between genotype and shear by a two-way ANOVA with Dunnett post-test; Figure 4(c-d)).

We next wanted to test whether elicited excitability is different for WT and G615E Na_V_1.5 channels. In current clamp mode, a -15-pA injection for 50 ms elicited a singular membrane potential spike in HEK-293 cells transfected with either WT or G615E Na_V_1.5 (Figure 5(a-b)). However, the shear-induced changes to the membrane potential spiking kinetics of WT Na_V_1.5 were not observed in cells with G615E Na_V_1.5 (Figure 5(b)). Since our data suggested an acceleration in Na_V_1.5 activation and inactivation with mechanical stress, we wanted to test whether submaximal electrical stimulation would result in increased electrical excitability in the presence of mechanical stimulation. Therefore, we designed a protocol that compared a step at maximal (fully activating) stimulation to a sequence of four steps at submaximal stimulation (Figure 5(c-d), *top traces*). At rest, we saw that at maximal stimulation (15 pA), >90% of steps led to spiking for both WT (98.3 ± 1.7%, n = 6, Figure 5(c,e)) and G615E Na_V_1.5 (90.8%±7.2%, n = 6, *P*> 0.05 *vs*. WT by a two-tailed non-parametric t-test; Figure 5(d,f)). Meanwhile, submaximal stimulation (10 pA) at rest led to spiking in a similar but reduced fraction of stimuli for both WT and G615E (WT, 23.4 ± 9.1% *vs*. G615E, 17.3 ± 4.6%; n = 6 each, *P*> 0.05 by a two-tailed non-parametric t-test). In the presence of shear stress, maximal stimulation continued to produce membrane potential spikes for >90% of depolarizations for both WT and G615E Na_V_1.5 (WT, 92.5 ± 4.8%; G615E, 92.4 ± 5.9%; n = 6 each, *P* > 0.05 by a two-tailed non-parametric t-test; Figure 5(c-f)), but only in WT and not in G615E did shear stress increase potentials induced by subthreshold stimuli (WT, 71.9 ± 14.4%; G615E 27.2 ± 8.5%; *P* < 0.05 WT control *vs*. WT shear and *P* < 0.05 interaction between stimulus, genotype, and shear by a three-way ANOVA with Tukey post-test).

Shear stress accelerated the spiking upstroke for WT Na_V_1.5, with the time from 10-pA stimulus to peak potential (t_PEAK_) decreasing from 51.3 ± 3.7 ms to 35.4 ± 2.6 ms (30.0 ± 5.2% faster) (Figure 5(g,i)). However, shear had no effect on G615E Na_V_1.5 t_PEAK_ (46.4 ± 3.1 ms to 41.0 ± 1.3 ms; 9.8 ± 5.8% faster) [n = 6 cells each; *P* < 0.05, WT control *vs*. WT shear; *P* > 0.05, G615E control to G615E shear; and *P* < 0.05, interaction between genotype and shear by a three-way ANOVA with Tukey post-test (g-h); and *P* < 0.05, WT to 0% change by a one-sample two-tailed t-test (I)] (Figure 5(h-i)). The effect of shear on either depolarization of the baseline (WT, +3.5 ± 3.6 mV *vs*. G615E, +4.2 ± 0.8 mV) or decrease in peak amplitude (WT, −3.9 ± 1.6 mV *vs*. G615E, −5.1 ± 2.5 mV) was not different. In all, the current-clamp data suggest that loss of mechanosensitivity in G615E Na_V_1.5 would lead to a loss of mechanically induced excitability in WT Na_V_1.5.

## Discussion

The goal of the current study was to examine Na_V_1.5 mechanosensitivity and its role in cellular mechano-electrical feedback. We used a unique *SCN5A* mutation G615E that is associated with acquired long-QT syndrome [19], sudden cardiac death [17], and irritable bowel syndrome [4]. In previous studies [4,17] and in this one, G615E Na_V_1.5 had mostly intact voltage-dependent function – normal voltage-dependence of activation, and either no or minor changes in voltage-dependence of inactivation.

On the other hand, we found dramatic differences in mechanosensitivity of voltage-dependent gating between WT and G615E Na_V_1.5. We tested both constructs in whole cell and patch, using shear stress and patch pressure as mechanical stimuli, respectively. For WT Na_V_1.5, we saw force produce several changes to voltage-dependent function, similar to previous studies on Na_V_1.5 [5,11,12,16,22] and Na_V_1.4 [23], but these changes were nearly absent for G615E Na_V_1.5. Thus, G615E Na_V_1.5 joins previously identified disease-associated mutations with abnormal Na_V_1.5 mechanosensitivity. These associated with long QT syndrome [13] in the heart and with IBS [5,6] in the gut and resulted in Na_V_1.5 with abnormalities in voltage-dependent function that were further accentuated by mechanical forces. However, to our knowledge, this is the first Na_V_1.5 mutation that has disrupted mechanosensitivity, while voltage-sensitivity remained mostly intact. Our findings may be relevant for understanding channelopathy mechanisms in cases when Na_V_1.5 mutations fail to reveal functional changes using voltage-dependence protocols. In such cases, and as we see with G615E Na_V_1.5, the functional impact may be on mechanical [6] or thermal [24] sensitivity.

Our results provide intriguing mechanistic insights on Na_V_1.5 mechanosensitivity. First, G615E is located on the intracellular linker connecting DI and DII, which is a novel location for a missense mutation to impact mechanosensitivity. Other Na_V_1.5 channelopathies like G298S have loss-of-mechanosensitivity and are in linkers as well. However, how these linkers contribute to the mechanism of Na_V_1.5 mechanosensitivity remains unclear. Second, our findings suggest that mechanisms of voltage- and mechano-sensation by Na_V_1.5 may be distinct. This is surprising since mechanical stimuli are well established to modulate voltage-sensitivity of voltage-gated channels [11,12,25] but not to introduce a separate mechano-gating paradigm [11,12]. Third, G615E Na_V_1.5 lost one but not both mechanosensitive responses – it lacked a perfusion-induced current increase but maintained a negative shift in the voltage-dependence of activation. This would suggest that mechanosensitive increases in peak Na^+^ current may be mechanistically distinct from mechanically induced shifts in voltage-dependence of activation and availability. Fourth, the findings suggest that the mechanosensitivity of the voltage-dependence of activation may be separate from that of availability. Previous and current studies show that mechanical stimuli accelerate the kinetics of activation and inactivation by the same constant. If the acceleration of activation by force is the rate-limiting step [26], it may explain the effect on the acceleration of inactivation. However, G615E Na_V_1.5 demonstrates an intact mechanosensitivity of activation but a loss of mechanosensitivity of inactivation, suggesting separate mechanisms. In all, our results shed important light on the mechanism of Na_V_1.5 mechanosensitivity and show that Na_V_1.5 mechano- and voltage-sensitivity may be targeted separately.

We are ultimately interested in understanding how Na_V_1.5 mechanosensitivity impacts SMC excitability, also called mechano-electrical feedback [8]. These cells undergo constant repetitive mechanical deformations – they are stretched during diastole and contracted during systole. Stretch is excitatory for WT Na_V_1.5 channels at the upstroke. But given the acceleration of inactivation, Na_V_1.5 stretch also results in a more significant current decrease during inactivation [11–13] and slowed recovery from inactivation [11]. Therefore, the overall impact of Na_V_1.5 mechanosensitivity on SMC function is difficult to judge only from the voltage-dependent operation. We designed current-clamp protocols in a reductionist system, a HEK-293 cell that expressed only WT Na_V_1.5 or G615E Na_V_1.5. Similar to previous studies [27,28], we found that these cells had both spontaneous and elicited electrical excitability. Compared to WT, G615E Na_V_1.5 had decreased mechanosensitivity of both spontaneous and elicited firing, suggesting that Na_V_1.5 mechanosensitivity may play an important excitatory role in SMC mechano-electrical feedback. However, a note of caution is required. We set the resting potential hyperpolarized to allow for full Na_V_1.5 availability, but this system limits our ability to determine whether sub-threshold events by either channel can result in firing, as may happen in excitable cells. In a set of preliminary studies, we investigated the potential influence of G615E-Na_V_1.5 on mechano-electrical feedback in GI SMCs by computational modeling. *In silico* modeling simulated Na_V_1.5 voltage- and current-clamp *in vitro* behavior, and the resulting SMC electrical activity and cytoplasmic Ca^2+^ concentrations were affected by mechanical forces for WT but not G615E-Na_V_1.5 (Supplementary Figure 2).

In addition to mechanosensitive voltage-gated sodium channels, cells have other mechanosensitive voltage-gated ion channels, such as potassium (K_V_) [29] and mechanically gated ion channels [30]. The effects of mechanical forces on these channels are expected to produce various electrical outcomes. For example, K_V_1.1 mechanosensitivity leads to “mechanical braking” of neuronal excitability [10]. Further, it will be important to determine the precise mechanical energies that the mechanosensitive ion channels encounter *in vivo* and to replicate these for studies *in vitro*. It is unclear how the current *in vitro* mechanostimulation protocols correlate with *in vivo* physiology. To fully understand mechano-electrical coupling we will need to integrate mechanosensitivity of Na_V_1.5 and other mechanosensitive ion channels into cell models and to place these models into physiologically relevant mechanical contexts.

In summary, the disease-associated Na_V_1.5 missense mutation G615E disrupts Na_V_1.5 mechanosensitivity without a significant impact on voltage-dependent function, which may have important consequences for mechano-electrical coupling in myocytes. This raises the possibility of directly targeting Na_V_1.5 mechanosensitivity in disease with a drug such as ranolazine [16,22], which does not have a significant effect on Na_V_1.5 peak current but can inhibit mechanosensitivity [15,16,31].
