# Supplementary material for: SCN5A mutation G615E results in NaV1.5 voltage-gated sodium channels with normal voltage-dependent function yet loss of mechanosensitivity
Source: Channels (Austin). 2019 Jul 2;13(1):287–98. doi: 10.1080/19336950.2019.1632670 (PMC6629189; doi:10.1080/19336950.2019.1632670)
Supplement: Supplemental Material [file kchl-13-01-1632670-s001.docx]

***SCN5A* mutation G615E results in Na_V_1.5 voltage-gated sodium channels with normal voltage-dependent function yet loss of mechanosensitivity:**

**SUPPLEMENTARY INFORMATION**

Peter R. Strege^1^, Arnaldo Mercado-Perez^1,3^, Amelia Mazzone^1^, Yuri A. Saito^1^, Cheryl E. Bernard^1^, Gianrico Farrugia^1,2^, Arthur Beyder^1,2*^

^1^Enteric NeuroScience Program, Division of Gastroenterology and Hepatology, ^2^Department of Physiology and Biomedical Engineering,

^3^Medical Scientist Training Program (MSTP), Mayo Clinic, Rochester, MN

Running Head: Abnormal mechanosensitivity in *SCN5A* mutation

*Corresponding author:

Arthur Beyder, M.D. Ph.D.

Mayo Clinic

200 First Street SW

Rochester, Minnesota 55905

Phone: 507-284-4695. Fax: 507-284-0266

Email: [beyder.arthur@mayo.edu](mailto:beyder.arthur@mayo.edu)

**SUPPLEMENTARY METHODS**

Modeling SMC mechanosensitivity *in silico*

***Whole-cell Na_V_1.5 current modeling in silico***. A Hodgkin-Huxley mathematical model was used to compose Matlab scripts (*MathWorks, Natick, MA)* to fit the experimental whole-cell WT and G615E Na_V_1.5 currents under no-shear and shear conditions. The model used 10 equations to describe the variation in time of the activating and inactivating gating variables *m* and *h*, respectively. As a result, there were a total of 15 arbitrary constants optimized to fit the experimental currents. For optimization, the *fminsearch* function was used with default tolerances (*TolFun =* 1e-4, *TolX* = 1e-4).

***SMC in silico.*** We performed experiments *in silico* with a modified SMC model^1^ to integrate Na_V_1.5 mechanosensitivity and examine its effects on SMC physiology, primarily for 3 reasons: (1) we required a separation of electrical and mechanical activation of Na_V_1.5; (2) SMCs likely have other mechanosensitive ion channels, and we did not currently want to determine their contributions; (3) we do not have access to SMCs with G615E Na_V_1.5.

The Corrias-Buist SMC model ^1^ from the CellML model repository (<https://www.cellml.org/>) ^2^ was employed to simulate a SMC. The TTX-resistant Na^+^ current was replaced with the WT Na_V_1.5 and G615E Na_V_1.5 currents recorded in this study. Equations developed to fit Na_V_1.5 currents in Matlab were used as the basis to describe sodium currents in the modified SMC model. An OpenCOR interface, along with a CVODE solver set to default tolerance (*Relative Tolerance = Absolute Tolerance =* 1e-07), was used to test and edit SMC simulations.

*In silico* voltage-clamp experiments were performed in a smooth muscle cell model modified from Corrias-Buist ^1^ by constraining δV/δt to 0. Voltage stimuli were the same ladders used in Matlab and whole cell experiments. The parameters derived for a channel clamped at -70 mV were used in a simulation that allowed the membrane potential to fluctuate over time. This simulation approximated the effect of mechanical stimuli on Na^+^ currents in smooth muscle and yielded estimates for Na^+^ currents and for dynamic changes in the membrane potential and free cytoplasmic calcium concentration.

**SUPPLEMENTARY FIGURES**

**Supplementary figure 1. Validation of modeling *in silico* of WT or G615E Na_V_1.5 currents recorded *in vitro*.**

*A-B*, Na^+^ current traces from whole-cell voltage clamp recordings or simulations of cells expressing WT (*A*) or G615E (*B*) Na_V_1.5 channels for voltage steps from -80 to -10 mV. *Control* and *shear* currents were normalized to the maximum peak current of control. *Difference* currents are the shear minus control currents. *In vitro* currents were averaged from 12 cells. *In silico* traces were derived with time constants calculated by fitting currents recorded in vitro. *Validation* currents are simulations in silico minus the composite currents in vitro. Negative deflections in the difference or validation currents indicate an acceleration of activation kinetics and/or an increase in peak current of shear or in silico, respectively. Positive deflections in the difference or validation currents indicate an acceleration of inactivation kinetics of shear or in silico, respectively.

*C-D*, Current-voltage (I-V) plots of peak Na^+^ currents from the traces shown in *A-B*, of WT (*C*) or G615E (*D*) Na_V_1.5 channels recorded in vitro (*black*) or simulated in silico (*red*) before (●) or during (○) shear stress.

**Supplementary figure 2. *In silico* simulation of smooth muscle cells.**

*A-B*, Na^+^ current traces from unconstrained simulations of cells coded with the -70 mV constants from WT (*A*) or G615E (*B*) Na_V_1.5 channels under control or shear conditions. Shear stress increased peak Na^+^ current of WT Na_V_1.5 *in silico* from -104 pA to -143 pA for +38% and of G615E from -84 pA to -101 pA for +20%.

*C*, Difference currents from the WT (*black*) or G615E (*red*) Na^+^ currents shown in *A-B*, after normalization to the peak current of either control. The shear-induced difference currents peaked at -62.7 pA for WT and -28.9 pA for G615E.

*D-E*, Membrane potential (V_m_) from unconstrained simulations of cells coded with the -70 mV constants from WT (*D*) or G615E (*E*) Na_V_1.5 channels under control or shear conditions. Shear stress accelerated the peak action potential in the model SMC with WT Na_V_1.5, from -33 mV at 20.4 ms to -32 mV at 17.6 ms, but not with G615E Na_V_1.5, from -35 mV at 22.3 ms to -36 mV at 22.3 ms.

*F*, Difference of the membrane potentials shown in *F-G* from model cells with WT (*black*) or G615E (*red*) Na_V_1.5 channels. The difference potential ranged from +6.1 to -7.2 mV for WT and +1.5 to -0.7 mV for G615E.

*G-H*, Intracellular free cytoplasmic calcium concentrations ([Ca^2+^_i_]) from unconstrained simulations of cells coded with the -70 mV constants from WT (*G*) or G615E (*H*) Na_V_1.5 channels under control or shear conditions. Shear increased and accelerated peak [Ca^2+^]_i_ in cells modeled with WT Na_V_1.5 from 1.02 µM at 25.9 ms to 1.07 µM at 22.8 ms for +5.6% peak and ‑12% time. Shear decreased peak [Ca^2+^]_i_ in cells with G615E Na_V_1.5 from 0.837 µM at 28.5 ms to 0.784 µM at 28.7 ms for ‑6.4% peak and +0.7% time.

*I*, Difference of the [Ca^2+^]_i_ concentrations shown in *G-H* from model cells with WT (*black*) or G615E (*red*) Na_V_1.5 channels, after normalization to peak [Ca^2+^]_i_ of either control. The difference concentrations of [Ca^2+^]_i_ in SMC increased by 42.7% with WT Na_V_1.5 and by 2.4% with G615E.

**SUPPLEMENTARY REFERENCES**

1. Corrias A, Buist ML. A quantitative model of gastric smooth muscle cellular activation. Ann Biomed Eng. 2007;35:(9):1595-607.

2. Lloyd CM, Lawson JR, Hunter PJ, Nielsen PF. The CellML Model Repository. Bioinformatics. 2008;24:(18):2122-3. 10.1093/bioinformatics/btn390
